# Tissue-Specific Transcriptomic Programs Coordinate Fruiting Body Formation and Development in *Grifola frondosa*

**DOI:** 10.3390/jof12070499

**Published:** 2026-07-08

**Authors:** Meiyan Zhang, Qiaozhen Li, Zhen Xu, Haoran Dong, Hailong Yu, Xiaodong Shang, Chunyan Song, Xiaoxia Song

**Affiliations:** Institute of Edible Fungi, Shanghai Academy of Agricultural Sciences, National Engineering Research Center of Edible Fungi, Shanghai 201403, China

**Keywords:** *Grifola frondosa*, fruiting body formation, RNA-Seq, tissue-specific expression, mitosis, meiosis

## Abstract

*Grifola frondosa* is a commercially important edible mushroom, yet the molecular mechanisms underlying fruiting body development in this species remain poorly understood. In this study, five sample types were collected from the same cultivation bag bearing mature fruiting bodies to represent different stages of a developmental continuum: spawn (S), white mycelial film (WM), gray mycelial mat (GM), fruiting body base (B), and pileus (P). Transcriptomic, biochemical, and morphological analyses were integrated to investigate the tissue-specific characteristics and potential roles of these tissues during fruiting body formation. The results revealed that S exhibited enhanced cellulase expression and activity, consistent with its proposed role in carbon supply for the developing fruiting body. WM showed upregulation of genes involved in H_2_O_2_ production and ATP synthesis, together with hydrophobin-related genes, consistent with active energy metabolism and a possible role in surface protection in this light-exposed layer. GM displayed distinct morphological features, including thicker hyphae with rounded tips and elevated expression of lectin, transcription-related, and signal transduction genes, consistent with its possible role as the site of primordium initiation. Both B and P specifically upregulated meiosis-related genes, suggesting their involvement in spore production. Collectively, these findings provide a comprehensive molecular resource for understanding tissue-specific genetic programs governing fruiting body formation in *G. frondosa*, while also highlighting candidate genes and testable hypotheses for future functional validation.

## 1. Introduction

*Grifola frondosa* is a widely cultivated edible mushroom recognized for its excellent sensory qualities and various pharmacological activities, including antitumor [1], antiviral [2], hypoglycemic, hypolipidemic [3], and gut microbiota-modulating effects [4,5]. In commercial production, a standardized cultivation process is typically employed: the mycelium is first allowed to fully colonize sawdust-based bags in darkness; then, light exposure induces the formation of a pigmented mycelial mat on the substrate surface (although the color may vary depending on the strain). Finally, primordia emerge from this mat and sequentially develop through a series of morphological stages, including the brain, post-brain, cauliflower, and cluster flower stages, before maturing into fruiting bodies [6]. Understanding the molecular mechanisms underlying these developmental transitions is crucial for improving yield stability, which remains a key challenge in the commercial cultivation of *G. frondosa*.

Transcriptomics has been widely used to study fruiting body development in edible mushrooms [7,8,9]. In *G. frondosa*, Wang et al. compared mycelia with primordia and found that primordium formation involved accelerated cell metabolism and increased protein synthesis [10]. Ren et al. analyzed four developmental stages, corresponding to primordium emergence, the brain stage, the post-brain stage, and mature fruiting bodies, as described by Barreto et al. [6], and observed that the latter three stages shared similar transcriptional profiles [11]. However, both studies focused on changes in either vegetative mycelium or already formed primordia and later developmental stages. The critical developmental window during which light exposure induces the formation of a pigmented mycelial mat on the substrate surface has not yet been investigated using transcriptomics.

Pigmented mycelial mat originates from the densification and pigmentation of the outer layer of the unpigmented vegetative mycelium, representing an interesting pre-fruiting phase of basidiomycete ontogenesis [12]. Its color shifts from light yellow to dark brown, and ultimately to black, with prolonged melanin accumulation, while its microstructure varies considerably across species [12]. Vetchinkina [12] described the dark orange mycelial mat of *G. frondosa* as homogeneous, less dense, and watery, consisting of tightly arranged pigmented cells with uniform content. In addition, this stage was characterized by a strong increase in melanin content, as well as high tyrosinase activity [12]. However, these characterizations have been limited to physiological and biochemical assays. Transcriptomic investigation is needed to uncover the molecular dynamics of the pigmented mycelial mat, a developmental checkpoint that directly influences subsequent primordium initiation.

To better investigate the pigmented mycelial mat, five sample types were collected from the same cultivation bag bearing mature fruiting bodies: spawn (S), white mycelial film (WM), gray mycelial mat (GM), base of the fruiting body (B), and pileus with sessile or short stipe (P). These samples represent a developmental progression from vegetative mycelium (S) through the pre-pigmented mycelial mat stage (WM) and pigmented mycelial mat stage (GM) to different tissues of mature fruiting bodies (B and P). Morphological observations were performed for all five sample types, including paraffin sections and scanning electron microscopy (SEM) of WM and GM. Biochemical assays and transcriptome sequencing were performed. Transcriptome analysis included clustering analysis to identify stage- or tissue-specific gene clusters and pairwise comparisons to detect differentially expressed genes between samples. In addition, the expression of five genes was validated by real-time quantitative PCR (RT-qPCR). These analyses allowed us not only to study the pigmented mycelial mat itself, but also to gain comprehensive molecular insights into fruiting body formation and development in *G. frondosa*.

## 2. Materials and Methods

### 2.1. Strain

The gray *G. frondosa* strain R134 was used as the experimental strain and was deposited in the China National Edible Fungi Germplasm Resource Bank (Shanghai) under accession number 7262.

### 2.2. Cultivation Methods

To prepare the substrate culture, strain R134 was first pre-cultivated in liquid medium at 24 °C in the dark for 9 days with shaking at 150 rpm. The liquid medium consisted of glucose (10 g/L), sucrose (10 g/L), soybean meal (2 g/L), corn flour (3 g/L), yeast extract (1.6 g/L), KH_2_PO_4_ (1 g/L), and MgSO_4_ (0.67 g/L). Subsequently, the pre-cultured liquid spawn was used to inoculate polypropylene cultivation bags (175 mm wide and 380 mm long), each containing 1.2 kg of sawdust-based substrate composed of 68% sawdust, 18% wheat bran, 7% corn flour, 6% soybean curd residue, 1% gypsum, with water added to adjust the moisture content to 65%. The bags were sterilized at 121 °C for 2 h. After cooling, each bag was inoculated with 25 mL of liquid spawn and incubated in the dark at 25 °C with a relative humidity of 60–70%.

After 40 days of mycelial culture, a circular hole (2.2 cm in diameter, approximately 3 cm from the inoculation point) was made on the side of each bag to induce primordium formation. The induction conditions were as follows: light intensity of 200–400 lx (12 h/d), CO_2_ concentration < 2000 ppm, temperature 18–20 °C, and relative humidity 85–90%. During the primordium growth stage, the environmental conditions were adjusted to 16–18 °C, CO_2_ ≤ 1500 ppm, and relative humidity > 85%. During pileus differentiation, the conditions were adjusted to 18–20 °C, CO_2_ ≤ 1000 ppm, and relative humidity > 95%. The fruiting bodies were harvested after 60 days of cultivation, with the harvest criterion being the disappearance of the marginal growth line of the pileus.

### 2.3. Sample Processing

**Figure 1:**
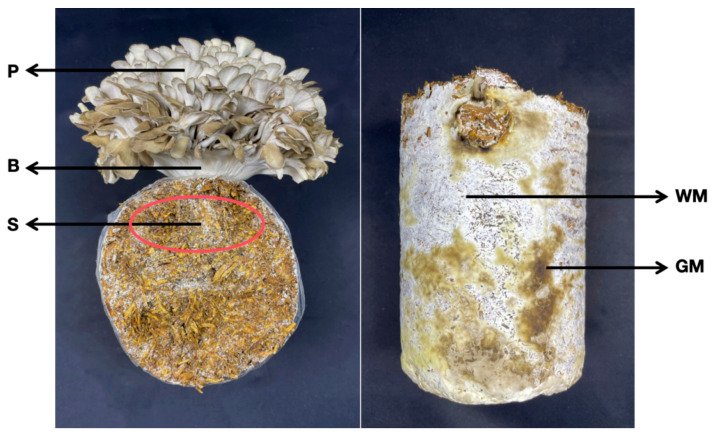
Schematic diagram of the sample types. S, spawn; B, base of the fruiting body; P, pileus; WM, white mycelial film; GM, gray mycelial mat. The red circle indicates the sampling area of S.

Five sample types were collected from different parts of the same cultivation bag (Figure 1). Sample S was collected from an area within 2–3 cm of the base of the fruiting body, whereas WM and GM were sampled from the bag surface. Three biological replicates were prepared for each sample type, with each replicate representing a pooled sample from three distinct cultivation bags. All samples were collected using sterile forceps, placed in sterile bags, immediately frozen in liquid nitrogen, and stored at −80 °C for subsequent analysis.

### 2.4. Morphological Observation

Morphological observations were performed on five sample types. To better visualize the structures, selected surface regions of S and pileus underside along with longitudinal sections of WM and GM were examined under a dissecting microscope. To further distinguish WM and GM, longitudinal paraffin sections were prepared for histological analysis, and both the outer and inner surfaces of WM and GM were examined by SEM.

#### 2.4.1. Paraffin Sectioning and Light Microscopy

WM and GM samples were fixed in FAA (70% ethanol: glacial acetic acid: 37% formaldehyde = 90:5:5, *v*/*v*/*v*) for 24 h at room temperature. After fixation, the samples were dehydrated through a graded ethanol series, cleared in xylene, and embedded in paraffin wax. Serial sections were cut at a thickness of 4 μm using a microtome and mounted onto glass slides. The sections were deparaffinized in xylene, rehydrated through a descending ethanol series, and stained with Safranin O for 2 h. After thorough rinsing, the sections were counterstained with Fast Green for 20 s, rapidly dehydrated through an ascending ethanol series, cleared in xylene, and mounted with neutral balsam. Histological observations were performed under a light microscope.

#### 2.4.2. SEM

WM and GM samples were prefixed in 2.5% glutaraldehyde for 2 h at room temperature and then stored at 4 °C. After rinsing three times with 0.1 M phosphate buffer (15 min each), the samples were post-fixed in 1% osmium tetroxide for 2 h at room temperature, rinsed again, and dehydrated through a graded ethanol series for 15 min per step. The samples were then critical-point dried with liquid CO_2_, mounted onto stubs with conductive tape, sputter-coated with gold–palladium, and examined under a scanning electron microscope.

### 2.5. Biochemical Index Detection

The levels of biochemical indices were measured using the corresponding assay kits following the manufacturer’s instructions. The soluble protein content, hydrogen peroxide (H_2_O_2_) content, exo-β-1,4-glucanase activity, and endo-β-1,4-glucanase activity were determined using a microplate reader. All kits were purchased from Suzhou Michy Biomedical Technology Co., Ltd. (Suzhou, China) with the following item numbers: soluble protein (M1805A), H_2_O_2_ (M0107A), exo-β-1,4-glucanase (M1722A), and endo-β-1,4-glucanase (M1733A). Each biochemical assay was performed with three biological replicates.

### 2.6. Transcriptomic Analysis

Transcriptomic sequencing and analysis were performed by Personal Biotechnology Co., Ltd. (Shanghai, China). Total RNA was extracted from each sample using pre-chilled TRIzol reagent, following the manufacturer’s instructions. The extracted RNA was used to construct cDNA libraries. For each sample, three independent sequencing libraries were constructed, and library quality was assessed using an Agilent Bioanalyzer 2100 (Agilent Technologies, Santa Clara, CA, USA). Sequencing was conducted on an Illumina NovaSeq 6000 platform in paired-end mode (2 × 150 bp). After filtering, the resulting clean reads were aligned to the *Grifola frondosa* reference genome downloaded from GenBank (BioSample accession: SAMN04531184) using HISAT2 v2.1.0 with default parameters.

Gene expression was quantified using HTSeq v0.9.1, and transcript abundance was normalized to FPKM values (fragments per kilobase of transcript per million mapped reads). Differentially expressed genes (DEGs) were identified using DESeq2 v1.38.3, with thresholds of |log_2_ (fold change)| > 1 and raw *p*-value < 0.05. Correlation analysis was performed using the Pearson’s correlation coefficient by default. Hierarchical clustering was conducted using the R packages pheatmap (version 1.0.12) and ComplexHeatmap (version 2.14.0), with Euclidean distance and the complete linkage method for bidirectional clustering.

To elucidate the functions of DEGs, Gene Ontology (GO) and Kyoto Encyclopedia of Genes and Genomes (KEGG) pathway enrichment analyses were performed using topGO v2.50.0 and clusterProfiler v4.6.0, respectively. DEGs were considered significantly enriched in GO terms or KEGG pathways at an adjusted *p*-value of <0.05.

Similar functional GO terms were aggregated according to their shared ancestor terms as identified in the ancestor chart available at https://www.ebi.ac.uk/QuickGO (accessed on 25 March 2026). Common DEGs shared among similar functional GO and KEGG terms were screened based on their gene IDs using Microsoft Excel 2010 (version 14.0).

### 2.7. RT-qPCR Validation

To validate the accuracy of the transcriptomic data, selected genes were subjected to RT-qPCR analysis using the same RNA samples used for transcriptomic sequencing. β-actin (β-actin-F: 5′-GAC ATG GAR AAG ATC TGG CA-3,’ and β-actin-R: 5′-TTC TCC TTG ATR TCA CGG ACR ATT TC-3′) [13] and 18S rRNA (18S-F: 5′-TGT TGT TGA TTA TCC TAA TTC-3,’ and 18S-R: 5′-CTT GGC TTT GAT ACT CTT-3′) [14] were used as internal reference genes. The details of reaction system and procedures were carried out as described by You et al. [15]. The 2^−∆∆CT^ method was adopted to calculate the relative expression levels of target genes. All data were obtained from three biological replicates, and each biological replicate included three technical replicates.

### 2.8. Statistical Analysis

Data were analyzed using SPSS 17.0, and results were expressed as the mean ± standard error of the mean. For intergroup comparisons, one-way analysis of variance was used, followed by Tukey’s honestly significant difference post hoc test, with homogeneity of variances assumed. A *p*-value < 0.05 was considered statistically significant.

## 3. Results

### 3.1. Morphological and Biochemical Characterization of Five Sample Types

**Figure 2:**
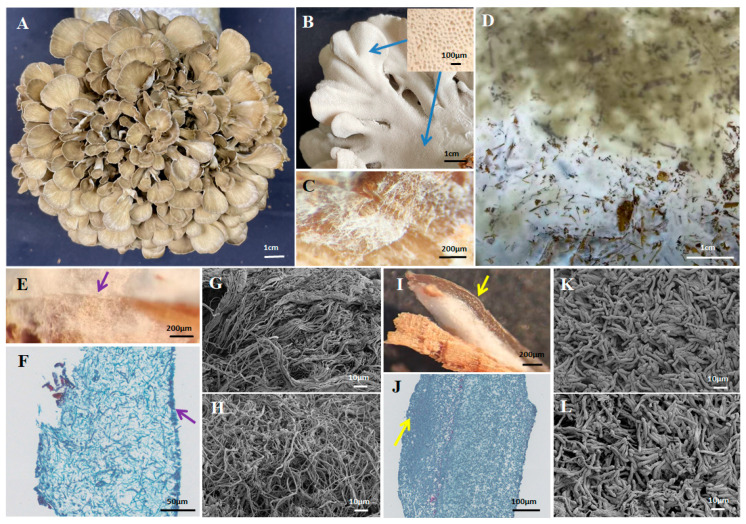
Morphological characteristics of the sample types. (**A**), top view of mature fruiting body; (**B**), pores on the pileus underside and base; (**C**), dissecting microscopic observation of mycelia growing on sawdust; (**D**), WM and GM; (**E**), oblique longitudinal sections of WM (dissecting microscopy); (**F**), longitudinal paraffin section of WM; (**G**), outer surface of WM (SEM); (**H**), inner surface of WM (SEM); (**I**), longitudinal sections of GM (dissecting microscopy); (**J**), longitudinal paraffin section of GM; (**K**), outer surface of GM (SEM); (**L**), inner surface of GM (SEM). Blue arrows indicate pores on the pileus underside and base, purple arrows indicate the outer surface of WM, and yellow arrows indicate the outer surface of GM.

The mature fruiting body of strain R134 resembled a blooming lotus flower (Figure 2A). The pilei were light grayish-brown and arranged in overlapping layers. The marginal pilei were fully expanded and fan-shaped, whereas the central pilei remained unexpanded and appeared funnel-shaped (Figure 2A). On the underside, dense white pores were present and extended to the B of the fruiting body (Figure 2B). S consisted of a mixture of mycelia and substrate. As shown by dissection microscopy (Figure 2C), the mycelia growing on sawdust were white and filamentous. On the substrate surface, WM was white, thin, and dry, whereas GM was gray, thick, and watery (Figure 2D). Further examination by dissection microscopy and paraffin sectioning revealed that both WM and GM consisted of two layers, with hyphae in GM being significantly thicker than those in WM (Figure 2E,F,I,J). Both layers of WM were white, with a thin outer layer of densely packed hyphae and a thicker but loosely arranged inner layer (Figure 2E,F). In GM, the outer layer was a thick aggregation of densely pigmented hyphae, whereas the inner layer was white, thicker, but less dense (Figure 2I,J). SEM further revealed distinct structural differences between WM and GM on both the outer and inner surfaces. WM exhibited slender hyphae on both surfaces, with the hyphae often forming bundles on the outer surface (Figure 2G,H). In contrast, GM displayed thicker hyphae on both surfaces, with numerous rounded hyphal tips (Figure 2K,L).

Physiological parameters, including soluble protein content, H_2_O_2_ content, and the activities of endo-β-1,4-glucanase and exo-β-1,4-glucanase, were also measured across the five sample types (Table 1). The soluble protein content was highest in GM, followed by P, B, S, and WM in descending order. H_2_O_2_ content was highest in S, lowest in B, and intermediate in P, GM, and WM. Regarding the activities of endo-β-1,4-glucanase and exo-β-1,4-glucanase, both were higher in S and WM than in B, P, and GM, with P showing the lowest activities among all types.

### 3.2. Transcriptomic Data

The uniquely mapped rates for S and WM (92–94%) were slightly lower than those for B, P, and GM (98–99%), which were attributable to the higher proportions of multiply mapped reads in the mycelial samples (Table S1). All samples exceeded the standard quality thresholds (uniquely mapped rate > 90%, Q30 > 95%), and FPKM normalization was applied to ensure reliable inter-sample comparisons. Moreover, the exon mapping rates exceeded 98% for all samples (Table S1), indicating that once the reads were mapped, they aligned to coding regions with equal efficiency across the sample types. Sequencing of the five sample types generated an average of 47,883,911 clean reads (Table S1).

**Figure 3:**
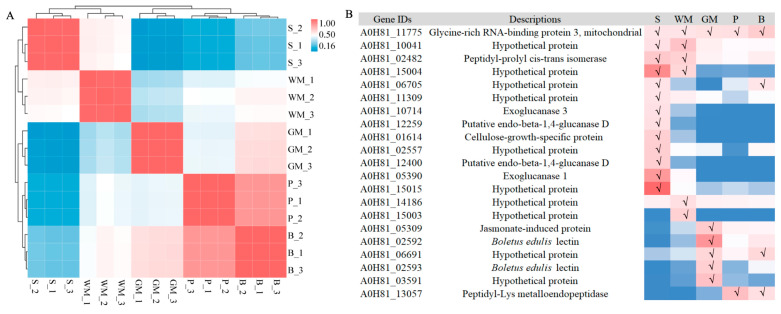
Heatmap of pairwise Pearson correlation analysis (**A**) and sample type-specific genes with FPKM > 10,000 (**B**) across five sample types. B, Colors from red to blue indicate FPKM values from high to low; checkmarks denote genes with FPKM > 10,000.

In total 11,939, 12,199, 12,213, 12,126, and 11,958 genes were detected in the S, B, P, GM, and WM samples, respectively, with the vast majority exhibiting FPKM values between 100 and 1000 (Table S2). Pairwise Pearson correlation analysis of gene expression profiles revealed that GM clustered with B and P, whereas WM formed a distinct branch with S (Figure 3A). Further analysis identified sample type-specific highly expressed genes (FPKM > 10,000) (Table S2; Figure 3B). Among these, only the genes with known functional annotations are described herein (Figure 3B): one gene encoding a glycine-rich RNA-binding protein 3 (all five types), one gene associated with peptidyl-prolyl cis-trans isomerase (S and WM), four genes related to cellulose degradation (S), one gene related to jasmonate-induced protein, two genes related to *Boletus edulis* lectin (GM), and one gene related to peptidyl-Lys metalloendopeptidase (B and P).

### 3.3. Clustering Analysis of DEGs and Functional Characterization of Each Cluster

**Figure 4:**
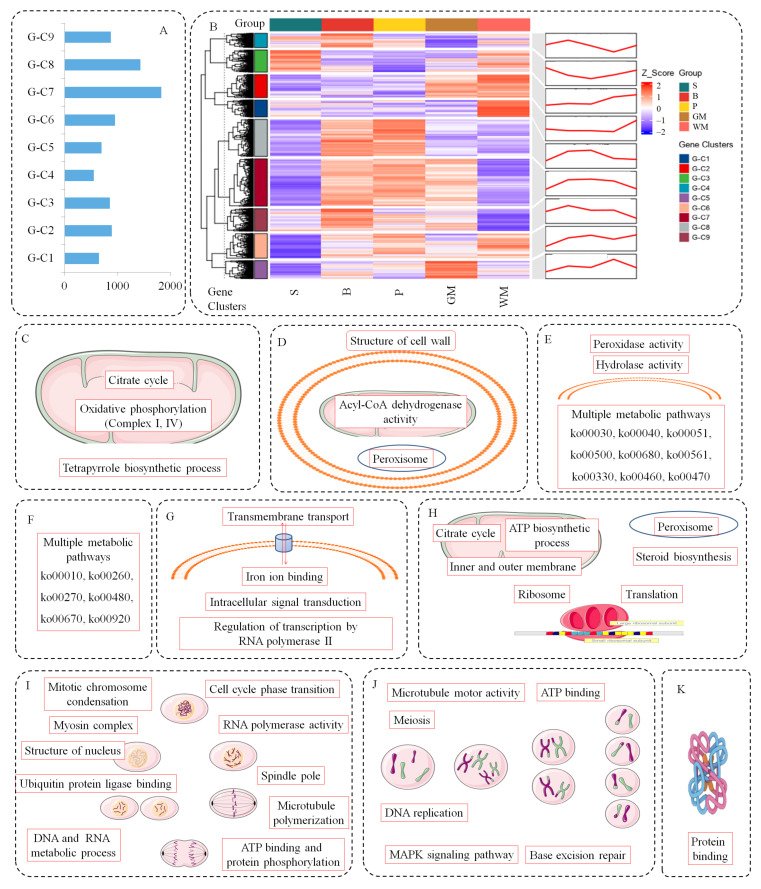
Number of DEGs (**A**), hierarchical clustering (**B**), and functional enrichment of DEGs (**C**–**K**) of nine clusters across five sample types. The background images in (**C**,**D**) were sourced from Bioicons (https://bioicons.com).

Cluster analysis was performed to identify groups of DEGs that exhibited similar expression dynamics across the five sample types. Based on expression similarities, 8699 DEGs were classified into nine distinct clusters, designated G-C1 to G-C9 (Data Set S1 Sheet S1; Figure 4A,B). DEGs from different clusters exhibited cluster-specific enrichment in GO and KEGG terms, as well as distinct expression patterns across the five sample types (Data Set S1 Sheets S1 and S2).

DEGs in G-C1 were predominantly upregulated in the WM and downregulated in the other four sample types (Figure 4B). This cluster was primarily associated with mitochondrial oxidative phosphorylation, with significant enrichment of Complex I (NADH dehydrogenase) and Complex IV (cytochrome-c oxidase), as well as the citrate cycle (ko00020) and tetrapyrrole biosynthetic processes (GO:0033014) (Figure 4C and Figure S1).

G-C2 DEGs were predominantly upregulated in both WM and GM but downregulated in S, B, and P (Figure 4B). Functional annotation revealed enrichment in oxidoreductase activity (i.e., acyl-CoA dehydrogenase), cell wall structure (i.e., hydrophobin-3, fruiting body protein SC3), and peroxisome (ko04146) (Figure 4D and Figure S2).

For G-C3, predominant upregulation was observed in S, followed by WM, whereas it was downregulated in GM, B, and P (Figure 4B). This cluster was notably enriched in hydrolase activity (especially cellulose-degrading genes), peroxidase activity (particularly lignin-degrading genes), and multiple metabolic pathways, including pentose and glucuronate interconversion (ko00040), the pentose phosphate pathway (ko00030), starch and sucrose metabolism (ko00500), fructose and mannose metabolism (ko00051), methane metabolism (ko00680), glycerolipid metabolism (ko00561), arginine and proline metabolism (ko00330), D-amino acid metabolism (ko00470), and cyanoamino acid metabolism (ko00460) (Figure 4E and Figure S3).

The DEGs of G-C4 were predominantly upregulated in B and downregulated in GM (Figure 4B). These genes were enriched in multiple metabolic pathways, including glycolysis/gluconeogenesis (ko00010); one-carbon metabolism by folate (ko00670), cysteine, and methionine metabolism (ko00270); glycine, serine, and threonine metabolism (ko00260); glutathione metabolism (ko00480); and sulfur metabolism (ko00920) (Figure 4F and Figure S4).

Turning to G-C5, this cluster exhibited predominant upregulation in GM and downregulation in S (Figure 4B). Associated functions included regulation of transcription by RNA polymerase II, iron ion binding, transmembrane transport, and intracellular signal transduction (GO:0035556) (Figure 4G and Figure S5).

DEGs from G-C6 were predominantly downregulated in the S group. (Figure 4B). This cluster was primarily associated with the ribosome and translation, mitochondrial inner and outer membranes, ATP biosynthesis, the citrate cycle, steroid biosynthesis, and the proteasome complex (Figure 4H and Figures S6 and S7).

DEGs of G-C7 were predominantly upregulated in GM, B, and P but downregulated in S and WM (Figure 4B). This cluster was associated with the structure of the nucleus (i.e., nuclear pore and envelope), mitotic chromosome condensation, DNA and RNA metabolic processes, RNA polymerase activity, ATP binding and protein phosphorylation (i.e., serine/threonine-protein kinase), cell cycle phase transition, ubiquitin protein ligase binding, spindle pole, microtubule polymerization, and myosin complex (Figure 4I and Figures S8 and S9).

G-C8 DEGs were predominantly upregulated in B and P but downregulated in S, GM, and WM (Figure 4B). Functional analysis indicated enrichment in meiosis, the MAPK signaling pathway, ATP binding (i.e., DNA replication licensing factors), microtubule motor activity, DNA replication and base excision repair (Figure 4J and Figure S10).

Finally, the DEGs of G-C9 were primarily associated with protein binding and exhibited predominant upregulation in B, whereas they were downregulated in WM (Figure 4B,K and Figure S11).

### 3.4. Comparative Analysis Between the Substrate-Inner Type (S) and the Substrate-Attached Surface Types (WM, GM and B)

**Figure 5:**
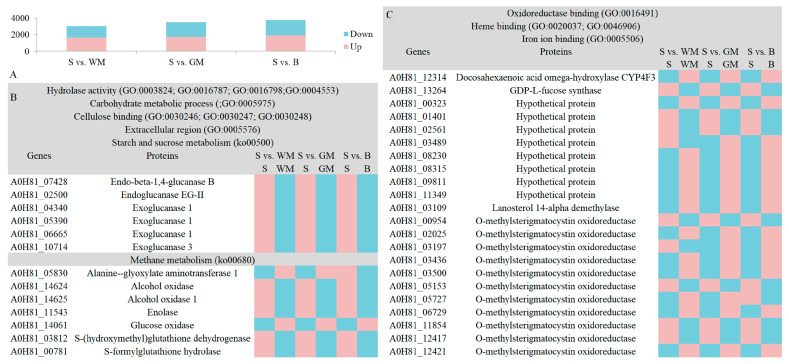
Number of DEGs (**A**) and shared GO and KEGG terms (**B**,**C**) in comparisons of S with WM, GM, and B. Red and blue denote upregulation and downregulation, respectively.

The comparison of S with WM, GM, and B generated three comparison pairs (S vs. WM, S vs. GM, and S vs. B) with distinct numbers of DEGs (Figure 5A). The DEGs were significantly enriched in specific GO and KEGG terms (Data Set S1 Sheet S3). Among these, 13 GO terms and two KEGG pathways were significantly enriched across all three comparison pairs (Figure 5B,C). These shared enrichments mainly involved three aspects: (1) hydrolase activity, carbohydrate metabolic process, cellulose binding, extracellular region, starch, and sucrose metabolism, mainly associated with cellulose-degrading genes (i.e., exoglucanase and endoglucanase); (2) methane metabolism; and (3) oxidoreductase, heme, and iron ion binding, mainly involving oxidoreductase genes (Figure 5B,C). Additionally, six GO terms and four KEGG pathways were enriched in two of the three comparative pairs (Data Set S1 Sheet S3).

**Figure 6:**
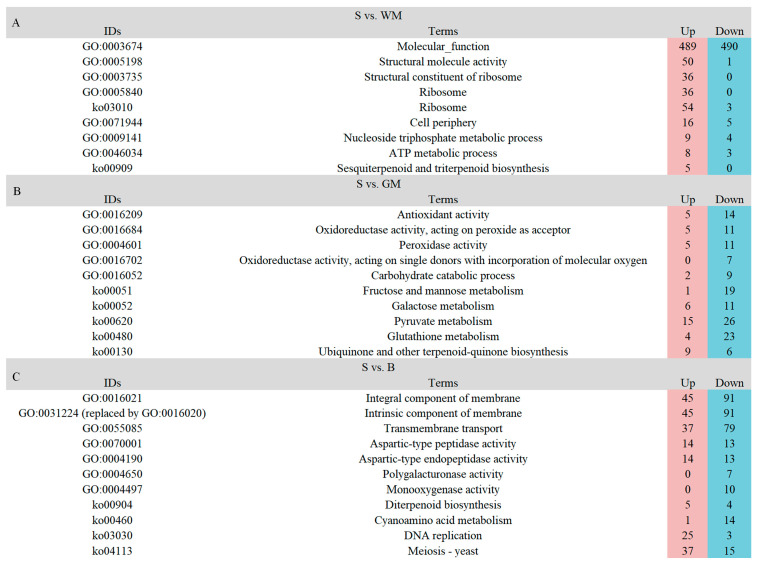
Distinct GO and KEGG terms (**A**–**C**) in comparisons of S with WM, GM, and B. Red and blue denote upregulation and downregulation, respectively.

Each comparison pair exhibited a distinct set of unique GO and KEGG terms (Figure 6A–C). In the S vs. WM comparison, seven GO and two KEGG pathways were enriched, including the ribosome (GO:0003674, GO:0005198, GO:0003735, GO:0005840 and ko03010), cell periphery (GO:0071944), ATP metabolic process (GO:0009141 and GO:0046034), and sesquiterpenoid and triterpenoid biosynthesis (ko00909) (Figure 6A). Among these, DEGs enriched in GO:0003735, GO:0005840, and ko00909 were upregulated (Table S3). In the S vs. GM comparison, five GO terms and five KEGG pathways associated with oxidoreductase activity (GO:0016209, GO:0016684, GO:0004601, and GO:0016702), carbohydrate metabolism (ko00051, ko00052 and ko00620), glutathione metabolism (ko00480), and ubiquinone and other terpenoid-quinone biosynthesis (ko00130) were screened (Figure 6B). The DEGs in GO:0016702 were downregulated (Table S4). For S vs. B, seven GO terms and four KEGG pathways were screened and linked to the following membrane components (GO:0016021 and GO:0031224): transmembrane transport (GO:0055085), aspartic-type endopeptidase activity (GO:0070001 and GO:0004190), polygalacturonase activity (GO:0004650), monooxygenase activity (GO:0004497), diterpenoid biosynthesis (ko00904), cyanoamino acid metabolism (ko00460), DNA replication (ko03030), and meiosis (ko04113) (Figure 6C). Among these, DEGs in GO:0004497 and GO:0004650 were downregulated (Table S5).

### 3.5. Comparative Analysis Across Developmental Stages and Fruiting Body Tissues (WM, GM, B and P)

**Figure 7:**
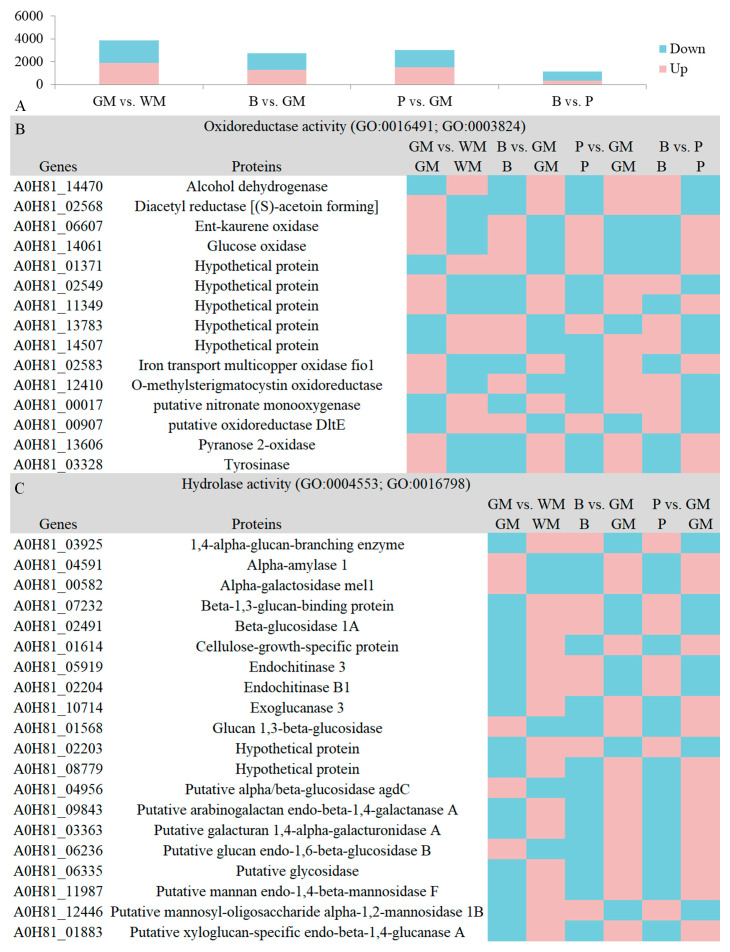
Number of DEGs (**A**) and shared GO and KEGG terms (**B**,**C**) in comparative analysis across developmental stages and fruiting body tissues (WM, GM, B and P). Red and blue denote upregulation and downregulation, respectively.

Four comparison pairs were generated: GM vs. WM, B vs. GM, P vs. GM, and B vs. P. The first three comparisons were associated with developmental stages, whereas the last revealed tissue-specific comparisons within the fruiting bodies. These four comparison pairs exhibited distinct numbers of DEGs as well as unique GO and KEGG terms (Figure 7A, Data Set S1 Sheet S4). Among these, two oxidoreductase activity-related GO terms (GO:0016491 and GO:0003824) were significantly enriched in all four pairs (Figure 7B). Two additional hydrolase activity-related GO terms (GO:0004553 and GO:0016798) were significantly enriched in the three comparison pairs associated with the developmental stages (Figure 7C). Furthermore, 13 GO and eight KEGG terms were enriched in two or three of the four pairs (Data Set S1 Sheet S4).

**Figure 8:**
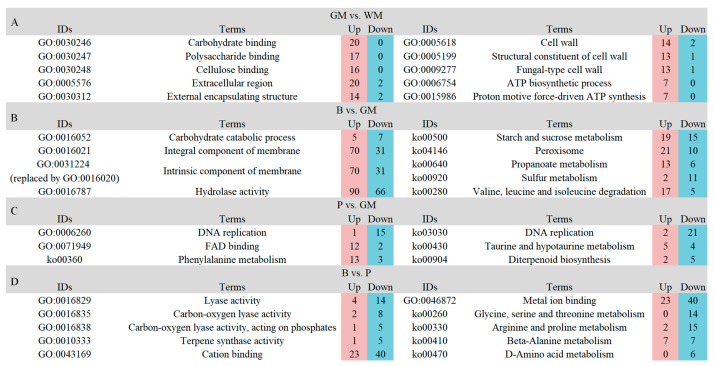
Distinct GO and KEGG terms (**A**–**D**) in comparative analysis across developmental stages and fruiting body tissues (WM, GM, B and P). Red and blue denote upregulation and downregulation, respectively.

Unique GO and KEGG pathways were identified for each pair (Figure 8A–D). The GM vs. WM generated ten enriched GO terms primarily involved in cellulose binding (GO:0030246, GO:0030247, and GO:0030248), cell wall (GO:0005576, GO:0030312, GO:0005618, GO:0005199, and GO:0009277), and proton motive force-driven ATP synthesis (GO:0006754 and GO:0015986) (Figure 8A). Among these, DEGs in GO:0030246, GO:0030247, GO:0030248, GO:0006754 and GO:0015986 were all upregulated (Table S6). B vs. GM showed four GO and five KEGG terms, including carbohydrate metabolism (GO:0016052, ko00500, ko00640); membrane components (GO:0031224, GO:0016021); hydrolase activity (GO:0016787); peroxisome (ko04146); sulfur metabolism (ko00920); and valine, leucine, and isoleucine degradation (ko00280) (Figure 8B). The P vs. GM comparison revealed two GO and four KEGG terms related mainly to DNA replication (GO:0006260, ko03030), FAD binding (GO:0071949), phenylalanine metabolism (ko00360), taurine and hypotaurine metabolism (ko00430), and diterpenoid biosynthesis (ko00904) (Figure 8C). B vs. P featured six GO and four KEGG pathways associated with terpene synthase activity (GO:0016829, GO:0016835, GO:0016838, GO:0010333), metal ion binding (GO:0043169, GO:0046872), amino acid metabolism(ko00260, ko00330), and other amino acids metabolism (ko00410, ko00470) (Figure 8D). Among these, the DEGs in ko00260 and ko00470 were downregulated (Table S7).

### 3.6. RT-qPCR Analysis of Selected Genes for Comparison with RNA-Seq Data

To provide complementary evidence for the transcriptomic data, five genes were selected for RT-qPCR analysis using β-actin and 18S rRNA as internal reference genes (Table 2, specific primers listed in Table S8). The qPCR results, normalized to β-actin and 18S rRNA, showed expression trends that were largely consistent with the FPKM values obtained from RNA-Seq. A0H81_05390 exhibited the highest expression level in S; A0H81_13057 and A0H81_09810 peaked in P; and A0H81_02592 and A0H81_01872 peaked in GM. Collectively, these results support the RNA-Seq expression patterns of the selected genes.

## 4. Discussion

Glycine-rich RNA-binding proteins (GR-RBPs) constitute a distinct subfamily of the glycine-rich protein superfamily and are extensively involved in the post-transcriptional regulation of RNA [16]. In studies on *Lentinula edodes*, Song et al. observed high expression of this gene in the culture substrate at different growth stages and in the pileus across various developmental periods [17,18]. Similarly, the present study detected high expression of this gene across all five sample types of *G. frondosa* (Figure 3B). These findings suggest that GR-RBPs play critical roles in the growth and development of edible mushrooms.

Accurate RT-qPCR quantification relies on the proper normalization of stably expressed reference genes. Yang et al. [19] found that the stability of internal reference genes varied across fungal species. In *G. frondosa*, β-actin and 18S rRNA are commonly used as reference genes [13,14]. To minimize the potential bias associated with single-gene normalization, we employed these two genes in parallel as dual internal controls. The comparable expression trends obtained with both reference genes not only support the reliability of our RT-qPCR results but also provide cross-validation for the expression patterns of the selected genes. However, one limitation of this study is that RT-qPCR validation was performed on only five representative genes. Given the thousands of differentially expressed genes identified by RNA-Seq, this small-scale validation did not constitute a comprehensive verification of the entire transcriptomic dataset. Further RT-qPCR analyses covering a broader range of genes would be valuable for substantiating the global expression patterns reported here. In addition, at the transcriptomic level, it should be acknowledged that the current conclusions were derived from a single strain (R134) at a single developmental stage (mature fruiting body). Therefore, the extrapolation of these findings to broader genetic backgrounds or developmental contexts should be performed with caution.

During fruiting body formation, the basal mycelium continuously supplies water and carbohydrates accumulated from the lignocellulose-rich substrate to the developing fruiting body [20,21]. Cellulases are a group of hydrolytic enzymes that degrade cellulose into glucose and serve as a primary carbon sources for various metabolic pathways [22]. Specific metabolites (i.e., soluble storage carbohydrates) can then be transported into the fruiting body to meet the demand for carbon skeletons, such as cell wall polysaccharides [17,22,23]. Ogawa et al. reported that the moisture concentration of the spawn increased rapidly within the approximately 2–3 cm region where the fruiting bodies of *L. edodes* developed [24]. In this study, sample type S corresponded to a similar region (Figure 1). In this region, mycelia of *G. frondosa* exhibited high gene expression and activity associated with both cellulases, endo-β-1,4-glucanase (carboxymethyl cellulase) and exo-β-1,4-glucanase (exoglucanase) (Figure 4E and Figure 5B, Table 1 and Table 2) [25]. Ni et al. also reported high carboxymethylcellulase activity in the substrate during fruiting body development in *G. frondosa* [26]. These results support, but do not prove, the possibility that S contributes to the carbon supply for fruiting body development.

H_2_O_2_ plays a key roles in both lignocellulose degradation and light-induced melanogenesis [17]. H_2_O_2_ accumulation in different *G. frondosa* tissues appears to be closely associated with their physiological functions: S penetrates the lignocellulose-rich substrate, whereas GM and WM cover the substrate surface; GM and P both undergo melanin accumulation, whereas B neither contacts the substrate nor accumulates melanin. Consistent with this, H_2_O_2_ content was the highest in S, lowest in B, and intermediate in P, GM, and WM (Table 1). The elevation in S likely resulted from enhanced peroxidase activities (Figure 4E), supporting lignin degradation within the substrate [17]. In the WM, some genes related to H_2_O_2_ production (i.e., complex I and acyl-CoA dehydrogenase) were upregulated (Figure 4C,D) [27,28], suggesting increased H_2_O_2_ content; however, the net level remained comparable to that in P and GM. This apparent paradox can be explained by the observation that oxidoreductase genes were differentially expressed across all seven pairwise comparisons; however, specific clusters varied among the samples (Figure 5C and Figure 7B). This indicates that each tissue possesses a distinct oxidoreductase repertoire. Thus, transcriptional upregulation in one tissue does not necessarily translate into higher H_2_O_2_ accumulation.

SEM characterization of the WM and GM surfaces in *G. frondosa* is reported here for the first time. Morphologically, WM consists of slender, often bundled, hyphae, whereas GM consists of significantly thicker hyphae with numerous rounded tips (Figure 2G,K). The rounded tips are likely associated with secretory activity during primordium formation, as such structures are rarely observed in WM. This morphological divergence is strongly supported by the transcriptomic data. In WM, genes involved in H_2_O_2_ production and ATP synthesis were predominantly upregulated compared with the other four sampling types (Figure 4B,C and Figure 8A), together with hydrophobin-related genes showing slightly higher expression in WM than in GM (Figure 4B,D). Hydrophobin-related genes and H_2_O_2_ production-related genes may contribute to surface protection in this light-exposed layer, whereas ATP synthesis supports the high-energy demand of such protective functions. In GM, elevated expression was observed for genes related to transcription by RNA polymerase II, transmembrane transport, and intracellular signal transduction (Figure 4B,G), together with specific upregulation of lectin genes (Figure 3B, Table 2). Lectins have been isolated from the mycelial mats of *G. frondosa* and play important roles in fruiting body formation [29]. Consistent with this, GM clustered transcriptionally with fruiting body tissues (B and P) rather than with WM (Figure 3A). Collectively, these results suggest that GM may represent an actively differentiated tissue predisposed to primordium formation, which is characterized by distinct morphological features, active secretion, and coordinated transcriptional regulation.

Fruiting body formation requires extensive cell proliferation and division through mitosis, which inherently creates a high demand for both new protein synthesis and chromatin remodeling [23]. Consistent with these requirements, GM, B, and P showed markedly upregulated genes encoding proteins involved in DNA transcription (DNA/RNA metabolic processes, RNA polymerase activity) [30] and mechanisms of chromosome congression during mitosis, including ATP binding, protein phosphorylation, cell cycle phase transition, mitotic chromosome condensation, spindle pole, and microtubule polymerization) (Figure 4I) [31]. Similarly, in *L. edodes*, mitosis-related genes have been reported to play key roles during primordium development [32]. Wang et al. [10] also reported an increase in protein synthesis during primordium development. In addition, the spore-producing region is the only site where meiosis occurs in the fruiting body. In *G. frondosa*, the spore-producing region is the tube layer, the distribution of which varies among different strains. In strain R134, the tube layer was distributed not only on the underside of the P tissue but also partially on the lateral side of the B tissue (Figure 2B). This explains why meiosis-related genes were specifically upregulated in the P and B (Figure 4J).

## Figures and Tables

**Table 1:** Comparison of soluble protein content, H_2_O_2_ content, and the activities of endo-β-1,4-glucanase and exo-β-1,4-glucanase across five sample types.

Sample Types	Soluble Protein Content (mg/g)	H_2_O_2_ Content (μmol/g)	Activity of Endo-β-1,4-glucanase (μg/min/g)	Activity of Exo-β-1,4-glucanase (μg/min/g)
S	0.3410 ± 0.0099 d	1.1416 ± 0.0243 a	100.1307 ± 0.8386 a	54.5965 ± 0.5659 b
B	0.4380 ± 0.0165 c	0.2361 ± 0.0117 c	21.5219 ± 1.0666 c	48.1504 ± 1.9992 c
P	0.6968 ± 0.0135 b	1.0030 ± 0.0164 b	8.1762 ± 0.4406 d	29.9533 ± 0.7729 d
GM	0.7724 ± 0.0050 a	0.9858 ± 0.0194 b	18.2674 ± 0.1339 c	26.1978 ± 0.5195 d
WM	0.2357 ± 0.0067 e	0.9969 ± 0.0166 b	84.4656 ± 1.2282 b	61.4900 ± 1.2250 a

All values are expressed based on fresh weight. Different superscript letters (e.g., a, b, c, d, e) indicate significant differences among groups, while the same letter indicates no significant difference.

**Table 2:** Comparison of RT-qPCR relative expression (two reference genes) and transcriptome FPKM values in five samples.

Gene IDs	Parameters *	S	B	P	GM	WM
AOH81_05390	I	33,280.82 ± 28.09 a	7.34 ± 0.21 c	6.65 ± 0.13 c	8.49 ± 0.81 c	4088.02 ± 32.59 b
	II	0.7167 ± 0.15 a	0 b	0 b	0 b	0.1267 ± 0.02 b
	III	1.0800 ± 0.08 a	0 b	0 b	0 b	0.0900 ± 0.01 b
AOH81_02592	I	21.42 ± 0.36 d	9583.47 ± 106.86 b	2906.62 ± 27.49 c	34,272.21 ± 625.62 a	1247.04 ± 24.26 d
	II	1.06 ± 0.13 d	376.87 ± 11.25 b	200.66 ± 46.34 c	2926.88 ± 257.27 a	140.60 ± 10.7 d
	III	1.0900 ± 0.07 c	147.0900 ± 7.5 b	40.3800 ± 1.18 c	881.7933 ± 33.67 a	44.66 ± 6.22 c
AOH81_13057	I	24.22 ± 1.94 d	12,014.41 ± 75.29 b	21,847.12 ± 17.31 a	876.21 ± 19.88 c	4.35 ± 1.10 d
	II	0.83 ± 0.12 b	265.03 ± 1.06 b	1027.52 ± 200.0 a	42.21 ± 1.42 b	0.46 ± 0.16 b
	III	1.11 ± 0.09 c	137.5 ± 6.44 b	265.31 ± 11.44 a	19.74 ± 0.92 c	0.16 ± 0.05 c
AOH81_09810	I	3.51 ± 0.38 d	688.38 ± 19.07 b	1470.93 ± 34.37 a	130.83 ± 9.77 c	37.53 ± 1.36 d
	II	0.78 ± 0.15 b	62.86 ± 1.55 b	171.40 ± 45.76 a	16.65 ± 1.69 b	25.21 ± 3.95 b
	III	1.13 ± 0.12 d	25.66 ± 0.79 b	36.17 ± 0.17 a	7.92 ± 0.29 c	9.37 ± 0.89 c
AOH81_01872	I	29.89 ± 0.37 d	251.53 ± 2.62 cd	382.80 ± 10.25 c	2904.23 ± 114.99 a	2107.16 ± 85.89 b
	II	0.76 ± 0.14 c	4.91 ± 0.12 c	16.05 ± 3.51 c	140.6 ± 14.66 a	86.64 ± 14.66 b
	III	1.05 ± 0.08 c	2.02 ± 0.07 c	3.21 ± 0.09 c	48.98 ± 2.96 a	31.07 ± 4.59 b

* I, FPKM from RNA-Seq; II and III, Relative expression levels determined by qRT-PCR (2^−∆∆CT^) normalized to β-actin and 18S rRNA, respectively. Different superscript letters (e.g., a, b, c, d) indicate significant differences among groups, while the same letter indicates no significant difference.

## Data Availability

The raw sequencing data were deposited in the NCBI BioProject database under accession number PRJNA1455280. Further inquiries can be directed to the corresponding authors.

## Supplementary Materials

The following supporting information can be downloaded at https://www.mdpi.com/article/10.3390/jof12070499/s1: Table S1: RNA-Seq data from five sample types; Table S2: Total gene numbers and gene counts within defined FPKM intervals for five sample types; Table S3: Upregulated DEGs enriched in GO:0003735, GO:0005840 and ko00909 (S vs. WM); Table S4: Downregulated DEGs enriched in GO:0016702 (S vs. GM); Table S5: Downregulated DEGs enriched in GO:0004497 and GO:0004650 (S vs. B); Table S6: Upregulated common DEGs enriched in GO:0030246, GO:0030247, GO:0030248, GO:0006754 and GO:0015986 (GM vs. WM); Table S7: Downregulated common DEGs enriched in ko00260 and ko00470 (B vs. P); Table S8: Primer sequences used for qPCR; Figure S1: Functional module classification, common DEG screening, and relationship prediction of significantly enriched GO and KEGG terms in G-C1; Figure S2: Functional module classification and common DEG screening of significantly enriched GO and KEGG terms in G-C2; Figure S3: Functional module classification and common DEG screening of significantly enriched GO and KEGG terms in G-C3; Figure S4: Functional module classification and common DEG screening of significantly enriched GO and KEGG terms in G-C4; Figure S5: Functional module classification and common DEG screening of significantly enriched GO and KEGG terms in G-C5; Figure S6: Functional module classification and common DEG screening of significantly enriched GO and KEGG terms in G-C6 (part 1); Figure S7: Functional module classification and common DEG screening of significantly enriched GO and KEGG terms in G-C6 (part 2); Figure S8: Functional module classification and common DEG screening of significantly enriched GO and KEGG terms in G-C7 (part 1); Figure S9: Functional module classification and common DEG screening of significantly enriched GO and KEGG terms in G-C7 (part 2); Figure S10: Functional module classification and common DEG screening of significantly enriched GO and KEGG terms in G-C8; Figure S11: Functional module classification and common DEG screening of significantly enriched GO and KEGG terms in G-C9. Data Set S1 Sheet S1: Enriched GO and KEGG terms of four comparative pairs between different developmental stages and fruiting body tissues(WM, GM, B and P); Data Set S1 Sheet S2: Significantly enriched GO and KEGG terms in the nine clusters; Data Set S1 Sheet S3: Enriched GO and KEGG terms of three comparative pairs between the substrate-inner site (S) and the substrate-attached surface types (WM, GM and B); Data Set S1 Sheet S4: Enriched GO and KEGG terms of four comparative pairs between different developmental stages and fruiting body tissues(WM, GM, B and P).
