# Supplementary material for: Tissue-Specific Transcriptomic Programs Coordinate Fruiting Body Formation and Development in Grifola frondosa
Source: J Fungi (Basel). 2026 Jul 8;12(7):499. doi: 10.3390/jof12070499 (PMC13413351; doi:10.3390/jof12070499)
Supplement: Supplementary file 1 [file jof-12-00499-s001.zip › Supplementary Materials.pdf]

Table S1. RNA-Seq data from five sample types

Types (Abbr.)	Samples	Raw reads (No.)	Clean reads (No.)	Multiple mapped (No.)	Multiple mapped rates (%)	Uniquely mapped (No.)	Uniquely mapped rates (%)	Mapped to exon rates (%)	Q20 (%)	Q30 (%)
Spawn (S)	S_1	46412216	45850204	3116192	7.35	31735116	92.22	98.28	98.90	95.23
	S_2	48403906	47816536	2737238	6.52	33362255	93.03	98.25	98.89	95.17
	S_3	39299228	38856148	1658083	4.29	26926382	92.05	98.37	98.94	95.32
Base of fruiting body (B)	B_1	41990408	41500210	582644	1.59	30391787	98.46	98.37	98.92	95.27
	B_2	51222554	50654088	585815	1.58	37060005	98.40	98.36	98.93	95.27
	B_3	37070488	36642266	588021	1.58	26815772	98.43	98.38	98.91	95.20
Pileus (P)	P_1	40345954	39859078	563843	1.66	29578934	98.45	98.44	98.89	95.18
	P_2	39490082	39046572	605347	1.61	28936555	98.45	98.45	98.92	95.26
	P_3	43789302	43315012	658576	1.70	32141430	98.44	98.43	99.05	95.81
Gray mycelial mat (GM)	GM_1	42707060	42233512	487489	1.56	30815680	98.44	98.41	99.06	95.94
	GM_2	46115490	45668860	543158	1.60	33430258	98.40	98.42	99.12	96.02
	GM_3	42160484	41712778	498702	1.61	30500331	98.39	98.42	99.06	95.85
White mycelial mat (WM)	WM_1	85618132	84293316	4550290	7.28	57939513	92.72	98.08	98.89	95.56
	WM_2	57716646	56737238	2675577	6.40	39137641	93.60	98.22	98.87	95.51
	WM_3	65169138	64072842	2813424	5.98	44258957	94.02	98.19	98.88	95.44
Mean values		48500739	47883911	1510960	3.49	34202041	96.23	98.34	98.95	95.47

Notes: Biological replicates are numbered 1 to 3 for each sample type.

.

Table S2. Total gene numbers and gene counts within defined FPKM intervals for five sample types

Sample types	Total genes (No.)	<1 (No.)	≥1 and <10 (No.)	≥10 and <100 (No.)	≥100 and <1000 (No.)	≥1000 and <10000 (No.)	≥10000 (No.)
S	11939	1729	4286	4714	1092	105	13
B	12199	1030	3166	6337	1551	111	4
P	12213	1138	3286	6092	1571	124	2
GM	12126	1339	3388	5818	1482	93	6
WM	11958	1362	3873	5012	1538	167	6

Table S3. Upregulated DEGs enriched in GO:0003735, GO:0005840 and ko00909 (S vs. WM)

GO:0003735/GO:0005840			
Genes	Proteins	Genes	Proteins
A0H81_11545	30S ribosomal protein S15, partial	A0H81_08363	54S ribosomal protein L4,
A0H81_00840	30S ribosomal protein S16	A0H81_12960	60S ribosomal protein L12
A0H81_03570	37S ribosomal protein MRP2, mitochondrial	A0H81_01154	60S ribosomal protein L15-B
A0H81_02073	37S ribosomal protein S8, mitochondrial	A0H81_00949	60S ribosomal protein L16
A0H81_04281	37S ribosomal protein SWS2, mitochondrial	A0H81_08789	60S ribosomal protein L17
A0H81_14461	40S ribosomal protein S13	A0H81_00627	60S ribosomal protein L22
A0H81_06059	40S ribosomal protein S15	A0H81_02199	60S ribosomal protein L23
A0H81_01034	40S ribosomal protein S16	A0H81_04024	60S ribosomal protein L3
A0H81_04253	40S ribosomal protein S21	A0H81_00756	60S ribosomal protein L32
A0H81_14463	40S ribosomal protein S6	A0H81_03344	60S ribosomal protein L34-A
A0H81_04021	40S ribosomal protein S7	A0H81_04069	60S ribosomal protein L35-3
A0H81_02967	50S ribosomal protein L14	A0H81_05839	60S ribosomal protein L37a
A0H81_00842	50S ribosomal protein L17	A0H81_09153	60S ribosomal protein L39
A0H81_03536	50S ribosomal protein L19	A0H81_02620	60S ribosomal protein L44
A0H81_10790	50S ribosomal protein L3	A0H81_08079	60S ribosomal protein L8
A0H81_08443	50S ribosomal protein L35	A0H81_09954	60S ribosomal protein L9-B
A0H81_08277	54S ribosomal protein L12, mitochondrial	A0H81_02466	Hypothetical protein
A0H81_10769	54S ribosomal protein L27, mitochondrial	A0H81_12980	Putative Na(+)/H(+) antiporter
ko00909			
A0H81_10954	Alpha-muurolene synthase	A0H81_07504	Squalene monooxygenase
A0H81_12697	Alpha-muurolene synthase	A0H81_06175	Squalene synthase
A0H81_06174	Hypothetical protein		

Table S4. Downregulated DEGs enriched in GO:0016702 (S vs. GM)

Genes	Proteins
A0H81_07304	1,2-dihydroxy-3-keto-5-methylthiopentene dioxygenase 1
A0H81_07305	1,2-dihydroxy-3-keto-5-methylthiopentene dioxygenase 1
A0H81_05293	Homogentisate 1,2-dioxygenase, partial
A0H81_07555	Homogentisate 1,2-dioxygenase
A0H81_04458	Hydroxyquinol 1,2-dioxygenase
A0H81_11903	Hydroxyquinol 1,2-dioxygenase
A0H81_12585	Hydroxyquinol 1,2-dioxygenase

Table S5. Downregulated DEGs enriched in GO:0004497 and GO:0004650 (S vs. B)

	GO:0004497		GO:0004650	
G	Genes	Proteins	Genes	Proteins
A	A0H81_05000	Baeyer-Villiger monooxygenase	A0H81_03399	Hypothetical protein
A	A0H81_08543	Electron transfer flavoprotein subunit beta	A0H81_11514	Putative exopolygalacturonase C
A	A0H81_12398	Flavin-containing monooxygenase FMO	A0H81_10111	Putative exopolygalacturonase X
A	A0H81_08720	Heme oxygenase 2	A0H81_12640	Putative exopolygalacturonase X
A	A0H81_12087	Inositol oxygenase	A0H81_03363	Putative galacturan 1,4-alpha-galacturonidase
A	A0H81_13541	Nitronate monooxygenase	A0H81_06686	Putative galacturan 1,4-alpha-galacturonidase
	A0H81_13893	Putative indole-3-pyruvate monooxygenase	A0H81_05725	Putative rhamnogalacturonase A
	A0H81_00017	putative nitronate monooxygenase		
	A0H81_04464	putative nitronate monooxygenase		
	A0H81_06271	putative nitronate monooxygenase		

Table S6. Upregulated common DEGs enriched in GO:0030246, GO:0030247, GO:0030248, GO:0006754 and GO:0015986 (GM vs. WM)

GO:0030246; GO:0030247; GO:0030248			
Genes	Proteins	Genes	Proteins
A0H81_10746	Cellobiose dehydrogenase	A0H81_06665	Exoglucanase 1
A0H81_01614	Cellulose-growth-specific protein	A0H81_10714	Exoglucanase 3
A0H81_08040	Endo-1,4-beta-xylanase C	A0H81_13264	GDP-L-fucose synthase
A0H81_11699	Endo-1,4-beta-xylanase D	A0H81_08779	Hypothetical protein
A0H81_07428	Endo-beta-1,4-glucanase B	A0H81_10141	Hypothetical protein
A0H81_02500	Endoglucanase EG-II	A0H81_01365	Putative acetylxylan esterase A
A0H81_04340	Exoglucanase 1	A0H81_11987	Putative mannan endo-1,4-beta-mannosidase F
A0H81_05390	Exoglucanase 1	A0H81_00735	Xyloglucanase
GO:0006754; GO:0015986			
A0H81_06473	ATP synthase subunit 4, mitochondrial	A0H81_01279	Hypothetical protein
A0H81_09065	ATP synthase subunit delta, mitochondrial	A0H81_10876	Hypothetical protein
A0H81_03048	ATP synthase subunit gamma, mitochondrial	A0H81_14239	Hypothetical protein
A0H81_11873	ATP synthase subunit O, mitochondrial		

Table S7. Downregulated common DEGs enriched in ko00260 and ko00470 (B vs. P)

Genes	Proteins
A0H81_02606	D-amino-acid oxidase
A0H81_01067	D-serine dehydratase
A0H81_01555	Hypothetical protein
A0H81_06772	Putative oxidoreductase YoxD

Table S8. Primer sequences used for qPCR

Gene	Forward primer sequence(5'to3')	Reverse primer sequence(5'to3')
A0H81_05390	TTCATCACCAACGACAAC	ATATTGACTACGGAGTTCTG
A0H81_02592	GCAGAGTATCAACAAGAC	TCAGGTTATGGTAATGGT
A0H81_13057	ATCTGCCTACTCACTCTT	TGTCTACTTGGATTCTGATG
A0H81_09810	TTCAACGCACTATTCACT	GCAGAAATAAAGGAAGCATA
A0H81_01872	GCAATGACGGGAATAACCAGTG	TCGGCGTGGTCGGATCAA

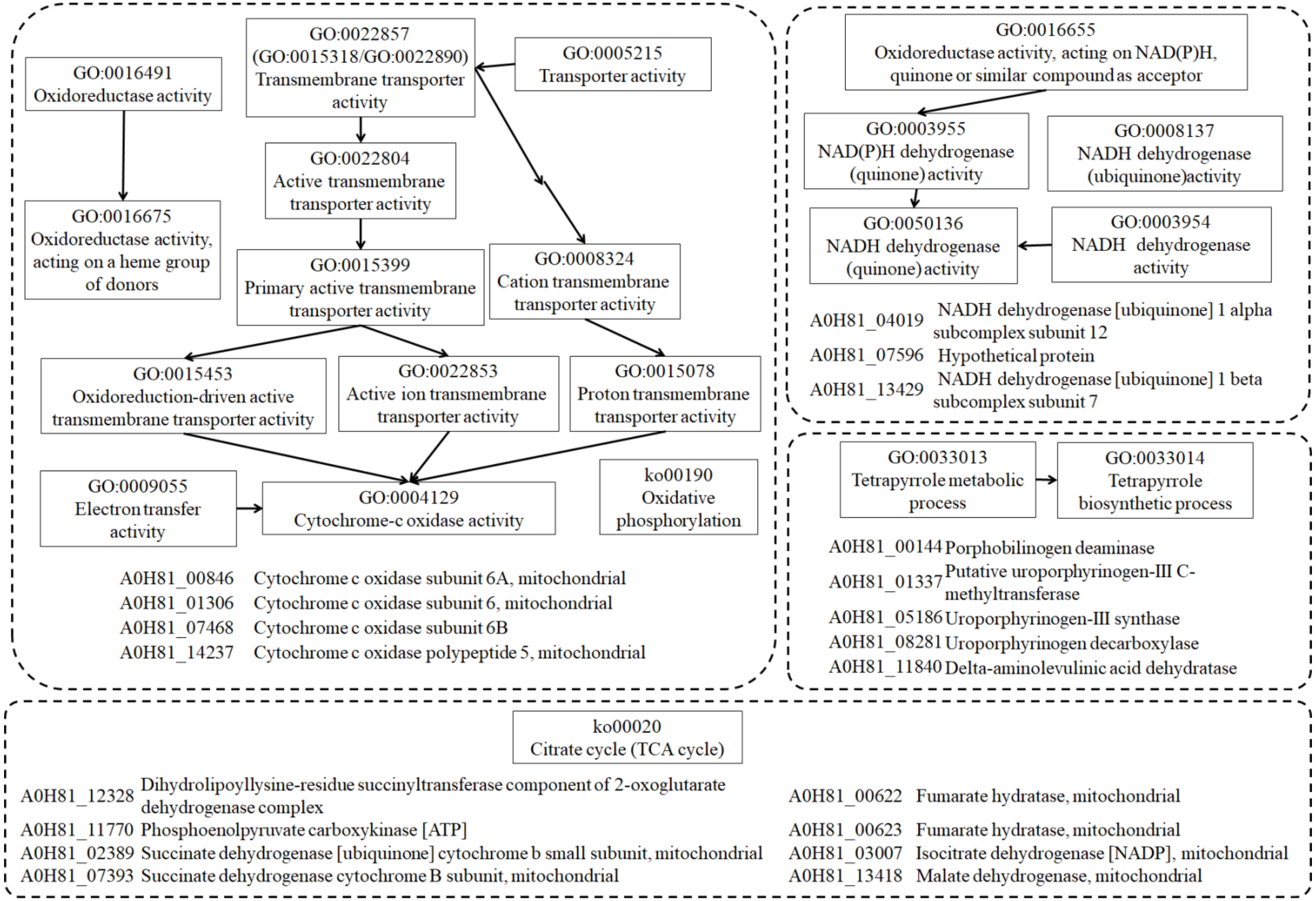

Figure S1 Functional module classification, common DEG screening, and relationship prediction of significantly enriched GO and KEGG terms in G-C1.

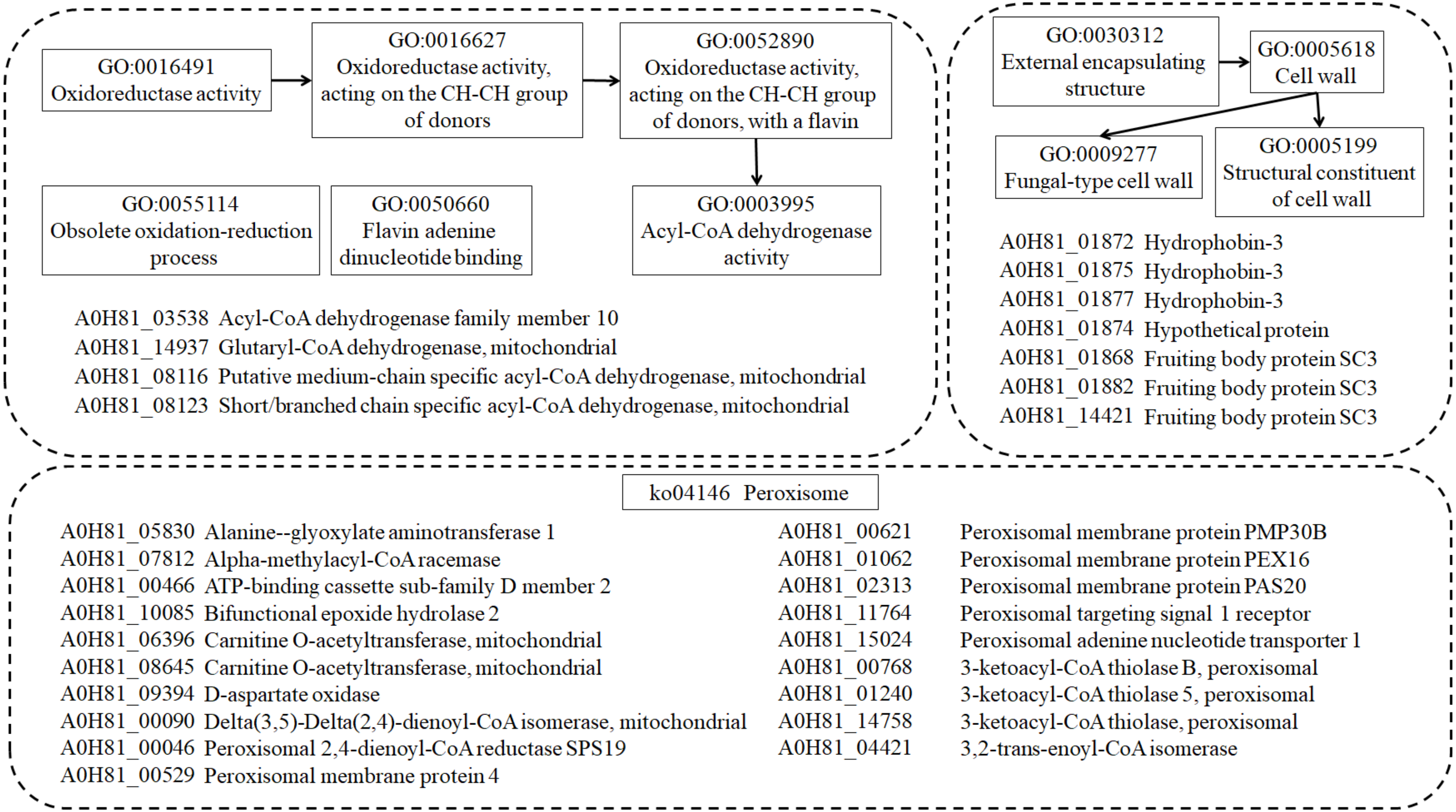

Figure S2 Functional module classification and common DEG screening of significantly enriched GO and KEGG terms in G-C2.

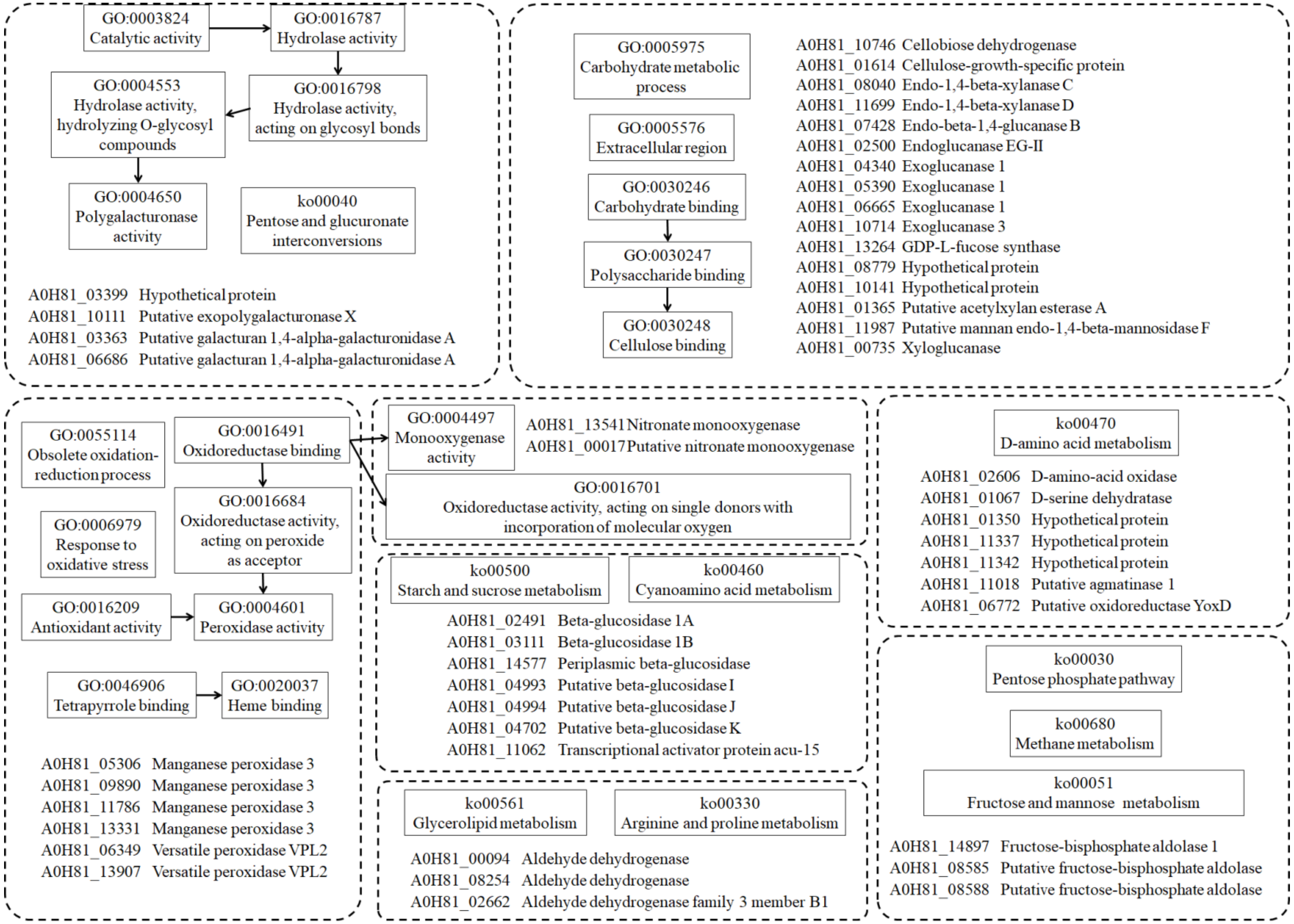

Figure S3 Functional module classification and common DEG screening of significantly enriched GO and KEGG terms in G-C3.

ko00010
Glycolysis/Gluconeogenesis

A0H81_11500 2,3-bisphosphoglycerate-independent phosphoglycerate mutase
A0H81_01754 Alcohol dehydrogenase 3
A0H81_07838 Aldehyde dehydrogenase family 3 member H1
A0H81_05382 Dihydrolipoyl dehydrogenase, mitochondrial
A0H81_11543 Enolase
A0H81_02087 Glucose-6-phosphate isomerase
A0H81_05461 Glyceraldehyde-3-phosphate dehydrogenase
A0H81_09008 Glyceraldehyde-3-phosphate dehydrogenase
A0H81_13950 Glyceraldehyde-3-phosphate dehydrogenase
A0H81_08052 Hypothetical protein
A0H81_08658 Pyruvate decarboxylase
A0H81_06956 Succinate-semialdehyde dehydrogenase [NADP(+)]
A0H81_02262 Triosephosphate isomerase

ko00260
Glycine, serine and threonine metabolism

ko00670
One carbon pool by folate

A0H81_02110 Cystathionine gamma-lyase
A0H81_02111 Cystathionine gamma-lyase
A0H81_05382 Dihydrolipoyl dehydrogenase, mitochondrial

A0H81_09324 Cysteine synthase 2
A0H81_07144 O-acetylhomoserine (thiol)-lyase
A0H81_14699 Protein TOXD
A0H81_03938 Putative serine-O-acetyltransferase cys2

ko00270
Cysteine and methionine metabolism

ko00920
Sulfur metabolism

ko00480
Glutathione metabolism

A0H81_00587 6-phosphogluconate dehydrogenase, decarboxylating 2
A0H81_13210 Cys-Gly metallodipeptidase dug1
A0H81_09075 Glutamate--cysteine ligase
A0H81_07382 Glutaredoxin-3
A0H81_04787 Glutathione reductase
A0H81_04843 Glutathione S-transferase 2
A0H81_02437 Hypothetical protein
A0H81_02706 Hypothetical protein
A0H81_06683 Saccharopine dehydrogenase [NADP(+), L-glutamate-forming]

Figure S4 Functional module classification and common DEG screening of significantly enriched GO and KEGG terms in G-C4.

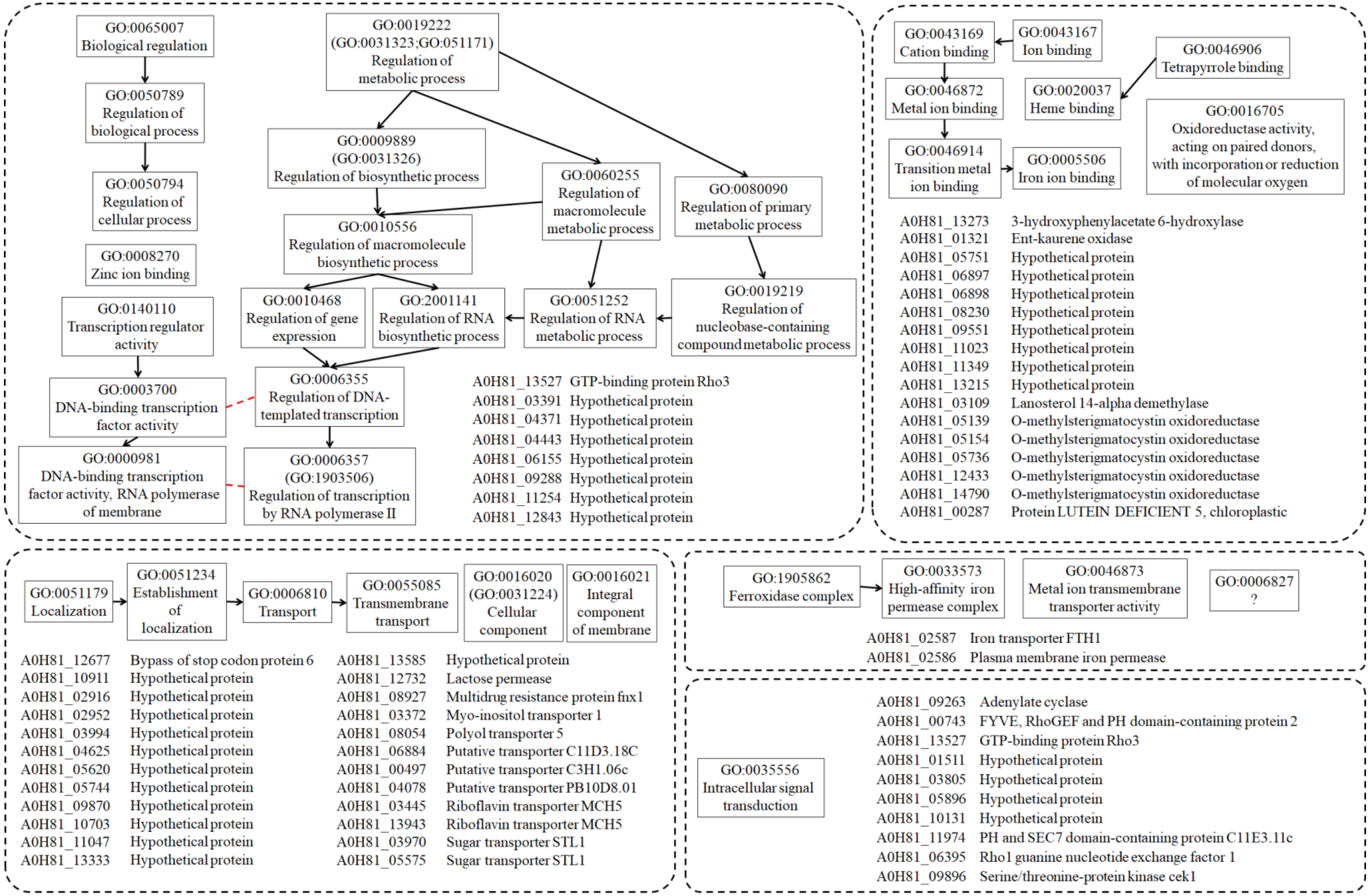

Figure S5 Functional module classification and common DEG screening of significantly enriched GO and KEGG terms in G-C5.

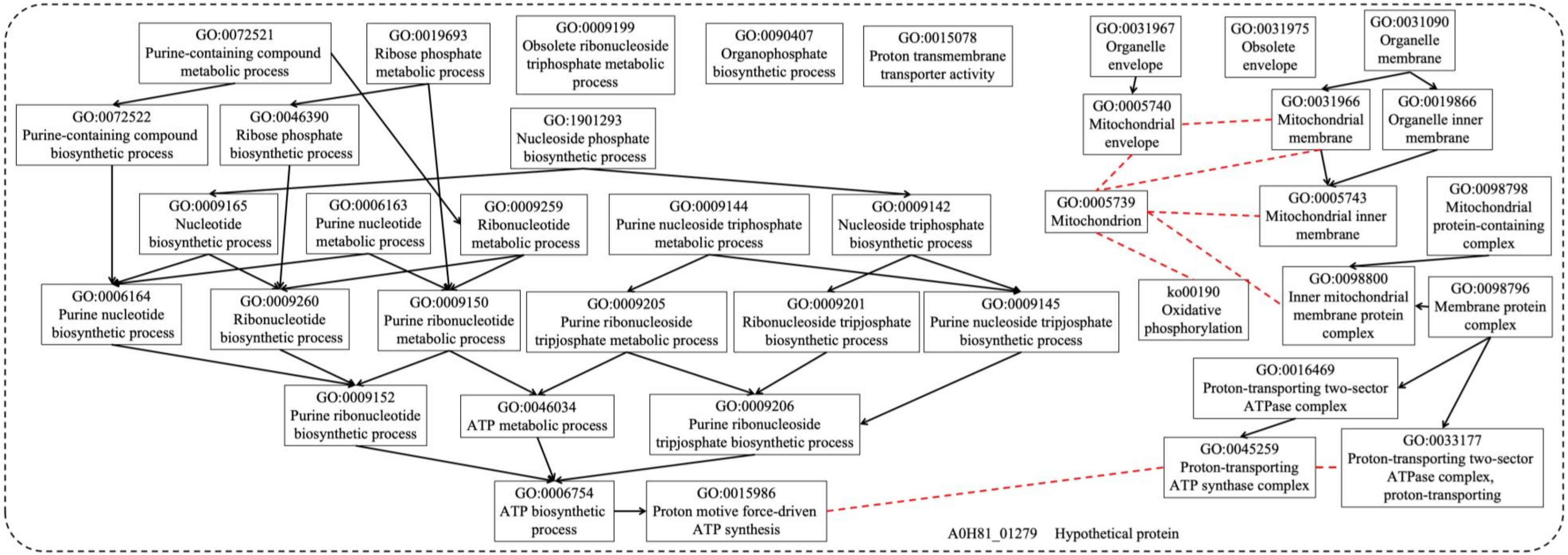

Figure S6 Functional module classification and common DEG screening of significantly enriched GO and KEGG terms in G-C6(part 1). Red dotted arrows indicate relationship prediction.

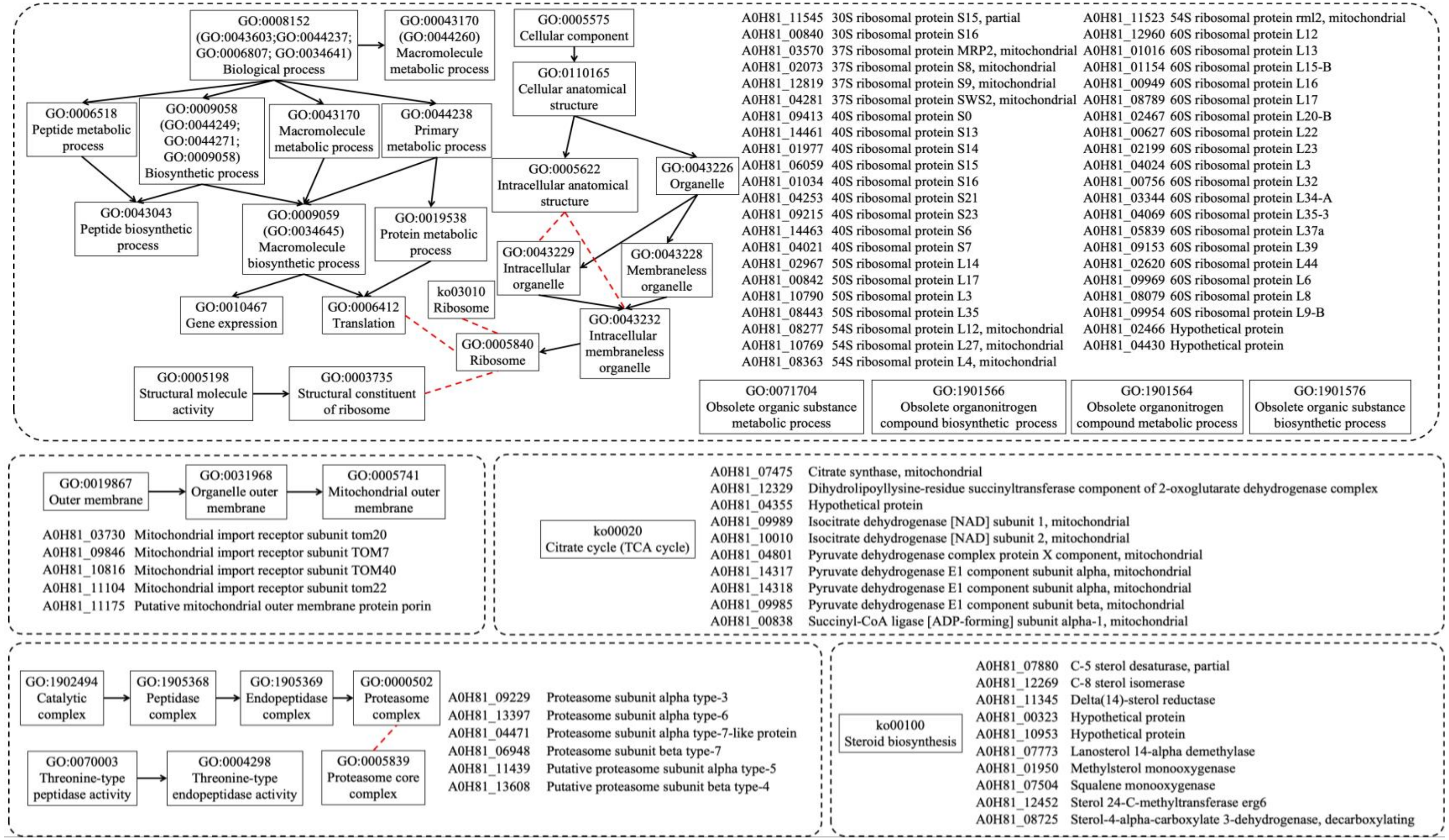

Figure S7 Functional module classification and common DEG screening of significantly enriched GO and KEGG terms in G-C6(Part 2).

Red dotted arrows indicate relationship prediction

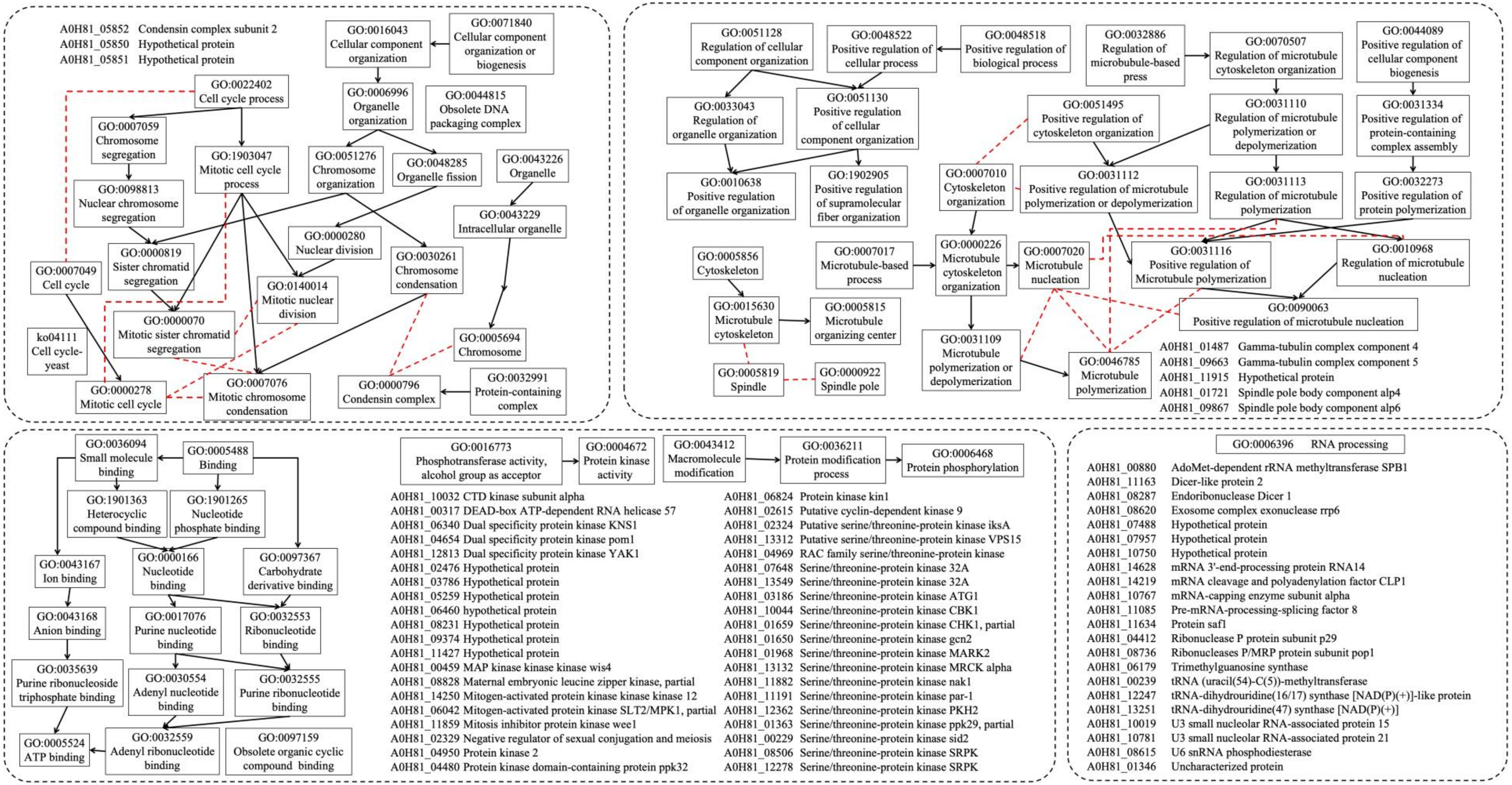

Figure S8 Functional module classification and common DEG screening of significantly enriched GO and KEGG terms in G-C7(part 1). Red dotted arrows indicate relationship prediction.

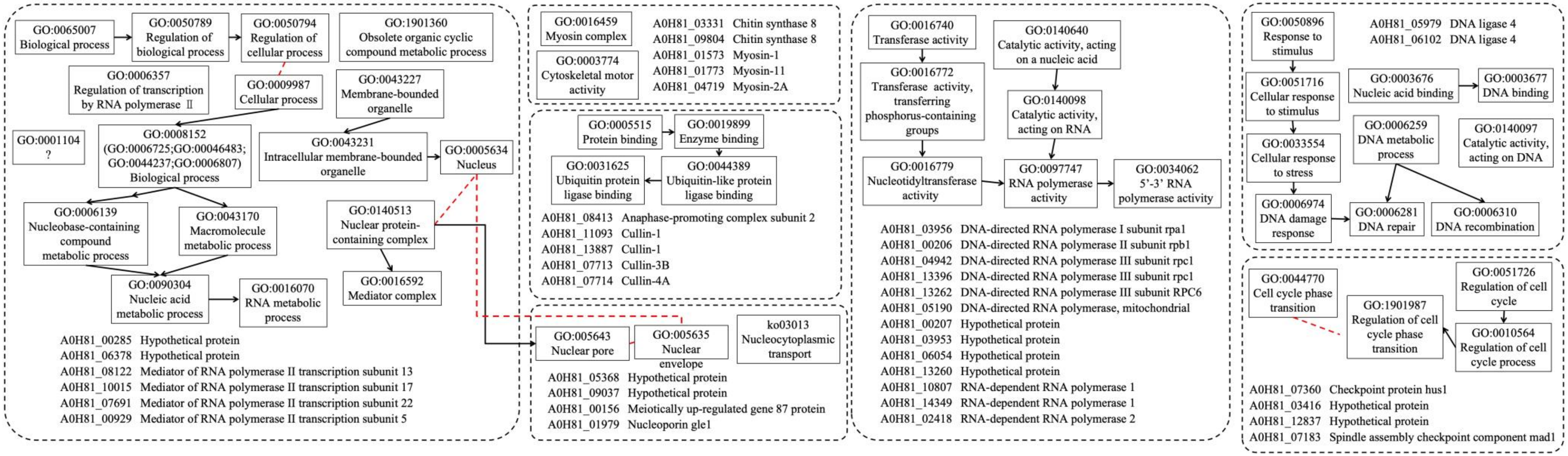

Figure S9 Functional module classification and common DEG screening of significantly enriched GO and KEGG terms in G-C7(Part 2). Red dotted arrows indicate relationship prediction.

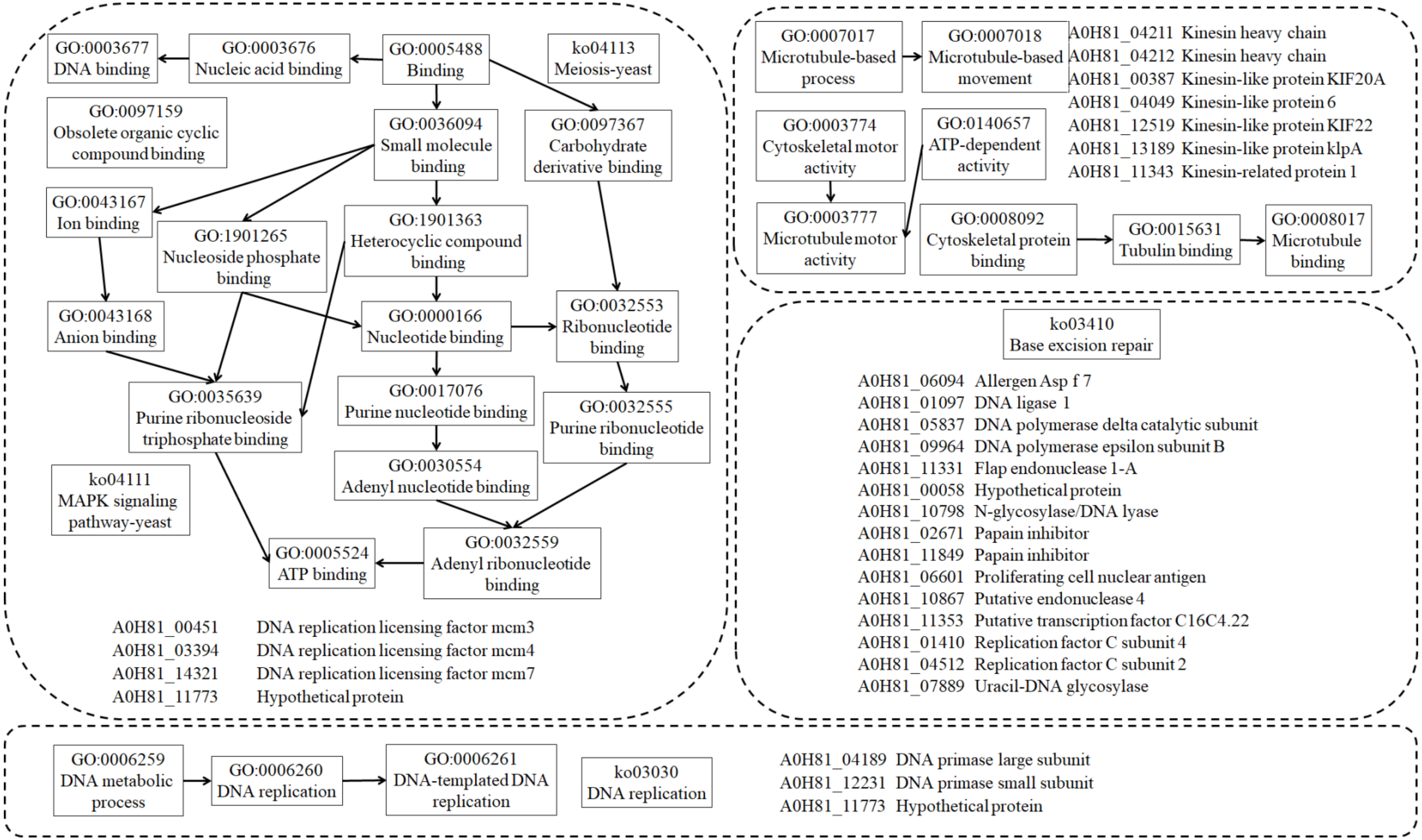

Figure S10 Functional module classification and common DEG screening of significantly enriched GO and KEGG terms in G-C8.

GO:0003674 Molecular function → GO:0005515 Protein binding

A0H81_11183	Alpha-actinin-like protein 1	A0H81_08764	Hypothetical protein
A0H81_04236	ATP-dependent molecular chaperone HSP82	A0H81_08851	Hypothetical protein
A0H81_11604	COP9 signalosome complex subunit 2	A0H81_09140	Hypothetical protein
A0H81_00241	Cullin-4	A0H81_10110	Hypothetical protein
A0H81_08719	DDB1- and CUL4-associated factor 5	A0H81_10880	Hypothetical protein
A0H81_14588	DnaJ subfamily C member 3	A0H81_10973	Hypothetical protein
A0H81_01766	E3 ubiquitin ligase BIG BROTHER-related protein	A0H81_11045	Hypothetical protein
A0H81_00556	E3 ubiquitin-protein ligase itt1	A0H81_11617	Hypothetical protein
A0H81_10314	Eukaryotic translation initiation factor 4 gamma	A0H81_11619	Hypothetical protein
A0H81_06536	F-box/WD repeat-containing protein 7	A0H81_12504	Hypothetical protein
A0H81_02287	Fimbrin	A0H81_12542	Hypothetical protein
A0H81_10150	Fizzy-related protein	A0H81_12696	Hypothetical protein
A0H81_10785	Glia maturation factor beta	A0H81_14035	Hypothetical protein
A0H81_08205	Heat shock protein sti1	A0H81_14409	Hypothetical protein
A0H81_06695	Histone-lysine N-methyltransferase SMYD1	A0H81_14734	Hypothetical protein
A0H81_00281	Hypothetical protein	A0H81_14736	Hypothetical protein
A0H81_01222	Hypothetical protein	A0H81_07021	Importin-alpha re-exporter
A0H81_01237	Hypothetical protein	A0H81_07910	Ingression protein fic1
A0H81_01299	Hypothetical protein	A0H81_11660	LEC14B protein
A0H81_02752	Hypothetical protein	A0H81_02501	Nucleoporin seh1
A0H81_02755	Hypothetical protein	A0H81_08274	Protein scd2/ral3
A0H81_03728	Hypothetical protein	A0H81_09868	Ras GTPase-activating-like protein rng2
A0H81_05205	Hypothetical protein	A0H81_09062	Receptor y region, transmembrane domain- and RING domain-containing protein 4
A0H81_06149	Hypothetical protein	A0H81_11927	Rho guanine nucleotide exchange factor scd1
A0H81_06190	Hypothetical protein	A0H81_07174	Serine/threonine-protein phosphatase T
A0H81_06249	Hypothetical protein	A0H81_01256	Small glutamine-rich tetratricopeptide repeat-containing protein beta
A0H81_06257	Hypothetical protein	A0H81_00910	Tetratricopeptide repeat protein 1
A0H81_06318	Hypothetical protein	A0H81_09086	UV excision repair protein rhp23
A0H81_07264	Hypothetical protein	A0H81_05017	Vegetative incompatibility protein HET-E-1
A0H81_07359	Hypothetical protein	A0H81_12090	Vegetative incompatibility protein HET-E-1
A0H81_08618	Hypothetical protein	A0H81_14455	Vegetative incompatibility protein HET-E-1

Figure S11 Functional module classification and common DEG screening of significantly enriched GO and KEGG terms in G-C9.
